# Validation of the Spanish version of the Pediatric Symptom Checklist (PSC) to identify and assess psychosocial problems among early adolescents in Chile

**DOI:** 10.1371/journal.pone.0283921

**Published:** 2023-04-06

**Authors:** Saray Ramírez, Sofía Gana, María Inés Godoy, Daniela Valenzuela, Ricardo Araya, Jorge Gaete

**Affiliations:** 1 Faculty of Education, Universidad de los Andes, Santiago, Chile; 2 Departamento de Evaluación, Medición y Registro Educacional, Universidad de Chile, Santiago, Chile; 3 Department of Psychology INVEST Research Flagship, University of Turku, Turku, Finland; 4 ANID, Millennium Science Initiative Program, Millennium Nucleus to Improve the Mental Health of Adolescents and Youths, Imhay, Santiago, Chile; 5 Department of Health Service & Population Research, King’s College London, London, United Kingdom; 6 David Goldberg Centre, Denmark Hill, London, United Kingdom; University of Valencia: Universitat de Valencia, SPAIN

## Abstract

**Background:**

The high prevalence of mental disorders in early adolescents, and their consequences, encourage the need for validated instruments to identify and assess psychosocial problems.

**Objectives:**

i) To evaluate the psychometric properties of the Spanish version of the Pediatric Symptom Checklist (PSC) questionnaires (PSC-Y, 35 items, and PSC-17-Y) and its subscales (Attention, Internalizing and Externalizing subscales), including the assessment of the item structure, concurrent validity, and reliability; and ii) To assess possible associations between bullying experiences, school climate and school membership with psychological problems identified by the PSC questionnaire.

**Methods:**

A cross-sectional study was carried out in 39 schools in Santiago, Chile. The sample consisted of 3,968 adolescents aged between 10 and 11 years. A descriptive analysis of the Pediatric Symptom Checklist was performed along with measures of dimensionality, reliability, and correlations with a validated questionnaire exploring similar constructs, the Strengths and Difficulties Questionnaire. Finally, associations of bullying, school climate, and school membership with the three subscales of the PSC were explored.

**Results:**

Both versions of PSC had problems with item #7 (Act as if driven by motor), which did not load in any of the latent factors. It was removed from later analyses. The three-factor structure of PSC was confirmed. All remaining items had high factor loadings in their corresponded latent factors, and the reliability was high for the total scales (PSC-34-Y, ω = 0.78; PSC-16-Y, ω = 0.94) and the subscales of PSC-16-Y (Attention, ω = 0.77; Internalizing, ω = 0.79; Externalizing, ω = 0.78). The goodness of fit was adequate, and the correlation between PSC subscales and SDQ subscales was high. Victimization and perpetration were associated with all PSC subscales, and higher school climate and stronger school memberships were negatively associated with PSC symptoms.

**Conclusions:**

The current findings seem to demonstrate that the Spanish version of the PSC is a valid and reliable instrument for identifying and assessing psychosocial problems in early adolescents.

## Introduction

Mental health problems among children and adolescents are prevalent and cause substantial disabilities over time [1,2]. Moreover, half of adult mental health disorders started their onset during adolescence [1,3]. Among adolescents, a meta-analysis published in 2015, which included data from 27 countries, reported a prevalence of 13.4% of psychiatric disorders [2], and a new meta-analysis published in 2020, collecting data from 19 countries, showed a prevalence of 31.0% [4]. The consequences of mental health problems in this population may have a lasting effect on their lives [5]. For instance, suicidal ideation and attempts are very frequent [6,7], and suicide is the second cause of mortality among young people [6,8]. Additionally, disability-adjusted life years (DALYs) among children aged 5–14 years old ranked second place among all causes of DALYs in the Americas and Europe [9], representing 10.7% and 11.4% of DALYs, respectively [9].

Another problem adolescents face today is the huge treatment gap found in different countries: most mental disorders become undetected, or there are many access barriers [10,11]. For instance, around 75% of children and adolescents with psychiatric disorders are not receiving treatment today [12]. Among these barriers, we can mention the lack of validated screening tools in several cultures, scarce implementation of their massive use, stigma and self-stigmatization [12–14], lacking of mental health infrastructure and services [11], few health professionals resources [11] and less than 1% of the health budget allocated to mental health in most low or middle-income countries [15].

Specifically, the availability and dissemination of instruments to identify psychosocial and behavioral problems [16] may help to reduce this gap. Indeed, several instruments are widely used to screen mental problems among adolescents [17–20], but many are not widely available in different cultures and contexts. Two recent systematic reviews found that the most commonly used instruments were the Child Behavior Checklist (CBCL), the Early Adolescent Temperament Questionnaire-Revised Version (EATQ-R), the Strengths and Difficulties Questionnaire (SDQ), Beck Youth Inventories (BYI), Behavior Assessment System for Children (BASC), Behavioral and Emotional Rating Scale (BERS), Child Health Questionnaire (CHQ), Child Symptom Inventories (CSI), and the Pediatric Symptoms Checklist (PSC) [17,18]. There are several advantages and disadvantages for each of them, but a detailed analysis of this issue is beyond the aims of this paper. However, we will provide information about some of these instruments to justify the necessity of validating the Pediatric Symptoms Checklist (PSC) in Chile.

The CBCL consists of 120 questions on problematic behaviors to be answered by the parents or carers of children aged 6–18 years old [21,22]. The current version of this questionnaire uses the algorithm of the Diagnostic and Statistical Manual of Mental Disorders (DSM-5) [21,22], and it is more specific about some disorders such as affective, anxiety, somatic, attention-deficit hyperactivity, oppositional defiant, and conduct problems. Among the disadvantages of this instrument are the extensiveness (which takes around 60 minutes to be answered) and that there is no self-reported version available for adolescents. The only validated version in Chile is for preschoolers (CBCL 1½-5) [23].

The EATQ-R measures the temperament and behavior of 15 years old adolescents [24,25]. It is available in self-report and parent-report formats. It has 103 items distributed in the following 13 subscales: activation control, activity level, affiliation, attention, fear, frustration, high-intensity pleasure, inhibitory control, perceptual sensitivity, pleasure sensitivity, shyness, aggression, and depressive mood [25]. In Chile, it has been validated among 12–18 years old adolescents [20]. However, it is a lengthy questionnaire, only two of the scales measure behavior problems, and we do not know the psychometric properties for a younger population (aged 9 to 11).

The SDQ consists of 25 items about emotional and behavioral problems in children and adolescents aged 4–16 [26]. It has three versions: self-reported, parents/carers-reported, and teachers-reported. It contains five subscales with five items each subscale: emotional symptoms, conduct problems, hyperactivity/attention problems, peer problems, and prosocial behavior [27]. The first four subscales are part of the total difficulties scale, while the last subscale is considered to be a part of the strengths [19]. In Chile, its psychometric properties have been studied with the parents/carers-reported version for children aged 4–11 years [28]. A recent validation study in Chile [19], conducted by the same research team, was conducted in a population of early adolescents (9–15 years old) with the self-report and parents/carers-reported versions. The main limitations found were related to the reliability of some of the subscales, especially for peer problems subscales (omega = 0.65, self-reported).

One important consideration when selecting an instrument is the availability of the psychometric properties of the scale for the age of the target population [29,30]. All of the studies carried out in Chile, except one [19], have this age limitation. Besides, most of the other studies showed less acceptable psychometric properties [20,23,28,31–34].

On the other hand, the PSC is an instrument that measures emotional and behavioral problems in children and adolescents between 4 and 18 years old [35]. The original questionnaire (PSC) has 35 items with a parent/carer-reported form, which showed good psychometric properties with high specificity (mean = 0.72) and sensitivity (mean = 0.88) values for identifying psychiatric disorders [36]. It has three subscales: attention, anxiety/depression (internalizing), and conduct (externalizing) [32]. Later on, a youth self-report questionnaire (PSC-Y, 35 items) was created. More recently, a shorter 17-item version for both parents (PSC-17) and youth (PSC-17-Y) [37] was produced based on the three original subscales, which were obtained from the three resulting factors [38]. This shorter version also showed good psychometric properties with an internal consistency test-retest of 0.85, for example [38]. This three-factor structure of the PSC provides evidence for the symptom clusterings of mental disorders initially proposed by Achenbach [39]: internalizing and externalizing, which currently are considered relevant to further incorporate the comorbidity concept in psychiatry [40].

In Chile, the first validation study was conducted over the parent version of the PSC 35-item [31] for first graders. This study found that two items ("absent from school and "worries a lot") were not reliable, so they were removed. The final version, the PSC-33-Spanish Chilean Version, has been widely used by the government of Chile and included as part of screening batteries of public programs, both in schools ("Life skills" program) [41] and in healthcare facilities [32]. Two other studies have used the parent-report format of the PSC-17 [32,33] but only among first graders. Therefore, no published studies have been conducted exploring the psychometric properties of the youth version of the Spanish version of the PSC-17-Y and PSC-Y, 35 items. Having self-reported information from adolescents may help to study the continuity of symptoms for those students who had participated in governmental programs when they were younger. Moreover, exploring the functioning of PSC-17-Y and PSC-Y, 35 items may give more options to professionals to select the suitable instruments according to population’ or patients’ necessities, measuring externalizing and internalizing problems, which appears to be a supported categorization to plan either prevention of treatment interventions [40].

Consequently, the aims of this study were: (1) to assess the validity of the internal factor structure of the subscales contained in the long (PSC-Y, 35 items) and brief (PSC-17-Y) young self-report questionnaires, (2) to explore the reliability of the subscales in both questionnaires, (3) to assess the concurrent validity of the resulting brief young self-report questionnaires with SDQ questionnaire; and (4) explore associations between several school risk and protective factors (bullying, school climate and sense of school membership) and the three subscales measured by the brief young self-report questionnaire.

## Materials and methods

### Study design

This is a cross-sectional study. This was part of a larger study aiming to assess the effectiveness of the antibullying program KiVa in Chile. Further details can be found in the clinical trial registration (ClinicalTrials.gov, NCT02898324)

### Setting and sample

The study population consisted of students attending 5th or 6th grade (ages 10–11) from a total of 39 schools located in Santiago, Chile.

The inclusion criteria of the participating schools were the following: (1) Schools that have 5th -6th grades, (2) mixed schools, (3) located in Santiago city, (4) having two classes per grade, and (5) High vulnerability (>75%) measured by the Chilean index School Vulnerability Index-National System of Equality Allocation (IVE-SINAE). This index is built every year by the Ministry of Education of Chile and assigned to every school based on poverty conditions such as poor health, low family income, receiving state benefits, and the risk of school failure of the students. In other words, the IVE-SINAE index shows the proportion of students in a school who are in most need [42,43]. All participating students are required to be fluent in Spanish in spoken and written language. Exclusion criterium included: students who had a pre-existence cognitive disability.

A total of 4,495 students were eligible to participate and were invited. Of these, a total of 3,968 (88.3%) consented and responded to the questionnaire. From this total sample, and for analytical purposes, it was decided to divide it into two samples through a simple random sampling method. The first sample (n = 1,981) helped to explore the psychometric properties of the PSC-Y, 35 items, and the second sample (n = 1,987) helped to explore the psychometric properties of the PSC-17-Y.

### Procedure and ethical considerations

The data was collected in November 2017 in accordance with the Declaration of Helsinki, with the approval of the ethics committee of the Universidad de los Andes (January 18^th^, 2016). First, school authorities were informed about the study aims, methods, and assessments, and afterward, they agreed to participate. Second, an informed and written consent form was sent to the parents or main caregivers in order to accept the participation of the students. Finally, informed and written assent was also provided by all students.

### Measures

#### Sociodemographic variables

We gathered the following information on sociodemographic factors: age (in years), gender (1 = Male; 2 = Female), grade class (1 = 5th grade; 2 = 6th grade), and School administrative dependency (1 = State school; 2 = Subsidized school). In Chile, around 93% of students attend state (40.4%) or subsidized (52.1%) schools [42].

#### PSC-Y, 35 item

The Pediatric Symptom Checklist youth self-report version is a questionnaire that helps identify and assess changes in emotional and behavioral problems in children and early adolescents. It has 35 items. Each item is rated as "*Never*", "*Sometimes*" and "*Often*" (scored 0, 1, and 2, respectively). Item scores are summed. It has three subscales, with the following respective items (numbers are indicated in relation to the PSC 35 items): Attention (#4 "Fidgety, unable to sit still", #7 "Acts as if driven by a motor") #8 "Daydreams too much", #9 "Distracted easily "and #14 "Has trouble concentrating". Anxiety/Depression or Internalizing subscale (#11 "Feels sad or unhappy", 13 "Feels hopeless", #19 "Is down on him or herself", #22 "Worries a lot" and #27 "Seems to be having less fun") and Conduct or Externalizing subscale (#16 "Fights with others", #29 "Does not listen to rules", #31 "Does not understand other people’s feelings", #32 "Teases others", #33 "Blames others for his or her troubles", #34 "Takes things that do not belong to him or her" and #35 "Refuses to share"). This instrument has been validated in several languages internationally and demonstrated good psychometric properties [35,37,38,44,45]. No validation of the Spanish version of this instrument is available in Chile.

#### PSC-17-Y

The PSC-17-Y is a brief youth self-reported questionnaire with a total of 17 items obtained from the three subscales of the youth self-reported 35-item version of PSC (PSC-Y). This version has also been validated [46] in several languages. No validation of the Spanish version of this instrument is available in Chile.

#### Strengths and Difficulties Questionnaire (SDQ)

The SDQ self-report questionnaire consists of 25 items aiming to assess emotional and behavioral problems among children and adolescents aged 4–17 years old [24]. It has five subscales with five items each: emotional symptoms, conduct problems, hyperactivity/attention problems, peer problems, and prosocial behavior [25]. The first four subscales are part of the difficulties scale, while the last subscale is considered the strengths scale [17]. It has good psychometric properties reported by its author [26] and in a Chilean validation study among adolescents [19]

#### Revised Olweus Bully/Victim Questionnaire

The Revised Olweus Bully/Victim Questionnaire (OBVQ-R) is a self-reported 40 items questionnaire that measures bullying and victimization in different ways, such as physical, verbal, racial, sexual, etc. [47]. This questionnaire has been validated and has good psychometric properties [48–50]. Three items from this questionnaire were used to assess associations of three kinds of bullying participation: victimization (Have you been bullied at school in the last two months?), perpetrator (Have you bullied a student at school in the last two months?), and bystanders (Have you watched or witnessed that a student has been bullied at school in the last two months?). These items were answered using a 5-point scale (1 = *Never* to 5 = *Several times a week*). For analytical purposes, all these answers were dichotomized (0 = *Never*; 1 = *Yes*).

#### The Psychological Sense of School Membership (PSSM)

PSSM is a self-reported instrument developed to assess the sense of school belonging. The original PSSM scale comprises 18 items: 13 positively worded statements and five negatively worded statements [51]. All of these items are related to students’ perceptions of being accepted, respected, included, and supported by others in the school social environment. In Chile, this instrument has been validated [52], resulting in a 13-item questionnaire containing 13 positively worded statements. For each statement, students answer on a 5-point scale (1  =  *not at all true*; 5  =  *completely true*). Therefore, a higher score means a higher sense of school membership.

#### School climate

This scale was used in the studies conducted in Finland to assess the effectiveness of the antibullying KiVa program [53], and it collects information about the perception of well-being by students at the school. Originally it had five items, but in the adaptation to Chile, four items had good psychometric parameters: "I feel safe at school", "I’m happy to be at my class", "I feel accepted when I am at the school" and "I like the school climate". For each statement, students answer on a 4-point scale (0 = *strongly disagree*; 4  =  *strongly agree*). Therefore, a higher score means a better school climate. For this particular sample, the scale psychometric properties were good, showing a Cronbach’s alpha of 0.84, and also the variance explained by items of the scale was 82.4%.

### Data analysis

#### Descriptive analysis

First, a descriptive analysis of sociodemographic variables was performed with measures of variance by calculating 95% confidence intervals and standard deviation for the age by class grade variable. In addition, a measure of central tendency was determined by calculating the mean age. Second, to represent the relative frequencies, percentages were calculated for the gender, class grade, and administrative school dependency variables. Third, a description of correlations of the PSC-Y and PSC-17-Y items and their respective subscales with the total score was given by Spearman’s Rho test with their respective p-value, where a figure of +1 indicates a perfect positive association, a figure of -1 indicates a perfect negative association and 0 indicates no association. Ranges of 0.70–0.99 (+/-) will be considered a strong association, 0.40–0.69 (+/-) a moderate association, and 0.01–0.39 (+/-) a weak association [54]. A p-value ≤ 0.05 will be considered for statistical significance. Finally, the resulting scale, tested and adapted from the PSC-17-Y, was analyzed, calculating the means, medians, standard deviations, kurtoses, skewnesses, and factor loadings. Factor loading >0.70 was considered optimal, 0.40–0.70 was considered moderate, and 0.30–0.39 was considered minimal [55].

#### Dimensionality and reliability analyses

The validity of the original versions of PSC-Y, PSC-17-Y, and the resulting versions PSC-34-Y and PSC-16-Y were tested using confirmatory factor analysis (CFA) with unweighted least squares (ULS) of the polychoric matrix due to the items ordinal distributions. The goodness of fit of the proposed models was evaluated using the following parameters: (1) the Root Mean Square Error of Approximation (RMSEA), where values below or equal to 0.05 were considered to be a good fit and values below or equal 0.08 were considered to be an acceptable fit [56,57]; (2) the Standardized Root Mean Square Residual (SRMR), where values below or equal to 0.10 were considered to be a good or acceptable fit [57]; (3) the Normalized Fit Index (NFI), where values above or equal to 0.95 were considered to be a good fit and values above 0.90 were considered to be an acceptable fit [58], (4) Non-Normed Fit Index (NNFI), where values above or equal to 0.97 indicates a good fit, and values above or equal 0.95 indicates an acceptable fit [59], (5) the Comparative Adjustment Index (CFI), where values above or equal to 0.97 were considered to be a good adjustment values above or equal 0.90 indicates an acceptable fit [57], (6) the Goodness of Fit Index (GFI), where values above or equal to 0.95 were considered to be a good fit and values above or equal to 0.90 were considered an acceptable fit [56]; and (7) the Adjusted Goodness of Fit Index (AGFI), where values above or equal to 0.90 were considered to be a good or an acceptable fit [56]. In addition, reliability was evaluated using the omega coefficient, where values of 0.65 or more are considered acceptable [60].

Concurrent validity was assessed between the three subscales of the resulting PSC-16-Y (attention, internalizing, and externalizing subscales) and the five subscales of the SDQ questionnaire (emotional problems, peer problems, conduct problems, hyperactivity problems, and prosocial behavior). This was evaluated with a Spearman correlation test [54]. A p-value ≤ 0.05 will be considered for statistical significance.

All dimensionality, reliability, and item descriptive analyses were performed in both instruments, PSC-Y and PSC-17-Y. We decided to present in the text the results of the PSC-17-Y, but all the analyses of the PSC-Y are included in the supplements.

#### Association analysis

We explored the associations of bullying participation, such as victimization, perpetration, and bystander, from the OBVQ-R questionnaire with the resulting PSC-16-Y, using the total score and subscale scores as dependent variables. We also explored the association of sense of school membership and school climate with the resulting PSC-16-Y. All of these dependent variables were analyzed as continuous variables, performing multivariable regression analyses and adjusting each model by gender and age. The unstandardized regression coefficient with 95% confidence intervals is presented, and the cutoff for statistical significance was established with a p-value < 0.05.

Descriptive analyses of the scales, internal structure statistical analyses, and concurrent validity analysis were conducted using R 3.5.0 software, and CFA was performed using the lavaan package in R 3.5.0. The significance level was stated at 0.05. Finally, sociodemographic descriptive analyses and association analyses were conducted using Stata 15.

## Results

### Description of the sociodemographic variables

A total of 3,968 students participated. 53,8% [52.3–55.4] were male, 52.1 [50.6–53.7] attended 6^th^ grade. The mean age was 9.8 (SD = 0.7) years old for 5^th^ graders and 10.8 (SD = 0.8) years old for 6^th^ graders. The students attended schools with different administrative dependencies: 50.1% [48.5–51.7] attended a state school, and 49.9% [48.3–51.5] attended a subsidized school. Both random samples had a similar distribution of sociodemographic variables (Table 1).

**Table 1:** Descriptive variables: Gender, class grade, age, and type of school.

	All sample			Sample 1			Sample 2		
Variable	N	% or Mean	[95%CI] or (SD)	N	% or Mean	[95%CI] or (SD)	N	% or Mean	[95%CI] or (SD)
Gender									
Female	1833	46.2	[44.6–47.7]	915	46.2	[44.0–48.4]	918	46.2	[44.0–48.4]
Male	2135	53.8	[52.3–55.4]	1066	53.8	[51.6–56.0]	1069	53.8	[51.6–56.0]
Class grade									
5th	1900	47.9	[46.3–49.4]	946	47.8	[45.6–50.0]	954	48.0	[45.8–50.2]
6th	2068	52.1	[50.6–53.7]	1035	52.2	[50.0–54.4]	1033	52.0	[49.8–54.2]
Age by class grade									
5th	1818	9.8	(0.7)	910	9.8	(0.8)	908	9.8	(0.7)
6th	1984	10.8	(0.8)	997	10.8	(0.8)	987	10.8	(0.8)
Administrative School dependency									
State	1988	50.1	[48.5–51.7]	992	50.1	[47.9–52.3]	996	50.1	[47.9–52.3]
Subsidized	1980	49.9	[48.3–51.5]	989	49.9	[47.7–52.1]	991	49.9	[47.7–52.1]

### Description of PSC-17-Y items

Most correlation coefficients between each item of PSC-Y and the PSC-17-Y with the total score of the respective subscale were moderate but significant. The only exception was found for the item "#7, Acts as if driven by motor", which did not correlate with the "Attention subscale" nor with the total score. This result was used to eliminate this item from the questionnaire. Later, we tested all psychometric properties for the resulting new subscales: "PSC-34-Y" and "PSC-16-Y". It is worth mentioning that the authors had authorization from the original authors of the PSC questionnaire to modify the questionnaire if needed (Table 2).

**Table 2:** Description of items correlations.

		PSC-Y		PSC-Y Factors		PSC-17-Y		PSC-17-Y Factors	
Subscale	Item	rho	p-value	rho	p-value	rho	p-value	rho	p-value
Attention	4. Fidgety, unable to sit still	0.46	0.00	0.48	0.00	0.45	0.00	0.49	0.00
Attention	7. Acts as if driven by a motor	-0.02	0.47	0.11	0.00	-0.01	0.72	0.08	0.00
Attention	8. Daydreams too much	0.42	0.00	0.34	0.00	0.41	0.00	0.33	0.00
Attention	9. Distracted easily	0.53	0.00	0.56	0.00	0.53	0.00	0.57	0.00
Attention	14. Has trouble concentrating	0.58	0.00	0.53	0.00	0.58	0.00	0.51	0.00
Internalizing	11. Feels sad, unhappy	0.54	0.00	0.55	0.00	0.47	0.00	0.56	0.00
Internalizing	13. Feels hopeless	0.56	0.00	0.56	0.00	0.53	0.00	0.58	0.00
Internalizing	19. Is down on him or herself	0.55	0.00	0.52	0.00	0.47	0.00	0.52	0.00
Internalizing	22. Worries a lot	0.44	0.00	0.41	0.00	0.44	0.00	0.44	0.00
Internalizing	27. Seems to be having less fun	0.52	0.00	0.48	0.00	0.45	0.00	0.50	0.00
Externalizing	16. Fights with others	0.45	0.00	0.43	0.00	0.48	0.00	0.48	0.00
Externalizing	29. Does not listen to rules	0.50	0.00	0.46	0.00	0.48	0.00	0.49	0.00
Externalizing	31. Does not understand other people’s feelings	0.35	0.00	0.33	0.00	0.39	0.00	0.39	0.00
Externalizing	32. Teases others	0.44	0.00	0.52	0.00	0.46	0.00	0.53	0.00
Externalizing	33. Blames others for his or her troubles	0.38	0.00	0.44	0.00	0.42	0.00	0.47	0.00
Externalizing	34. Takes things that do not belong to him or her	0.37	0.00	0.45	0.00	0.41	0.00	0.45	0.00
Externalizing	35. Refuses to share	0.36	0.00	0.41	0.00	0.35	0.00	0.41	0.00

Note: Rho value significance: null (0); weak (0.01–0.39); moderate (0.40–0.69); strong (0.70–0.99); perfect (1).

### Descriptive Statistics and factor loading of PSC-16-Y items

**Fig 1:**
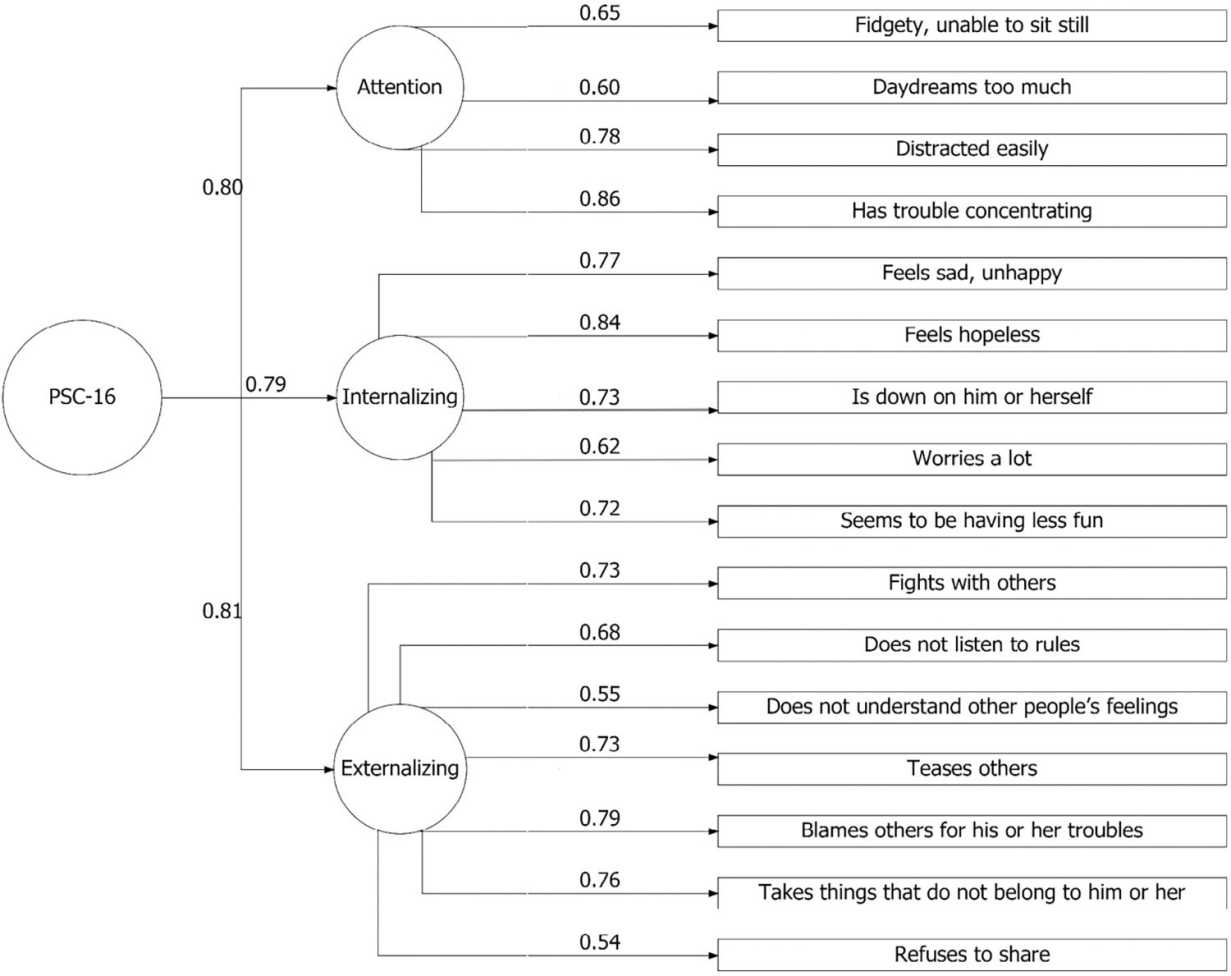
Confirmatory factor analysis of the PSC showing the three-factor structure.

All items had moderate to optimal factor loading figures. The higher mean scores were found for items from the Attention (# 4, Fidgety, unable to sit still; # 9, Distracted easily) and Internalizing subscales (# 13, Feels hopeless; (# 27, Seems to be having less fun) (See Table 3 and Fig 1).

**Table 3:** Descriptive Statistics and factor loading of the PSC-16-Y items.

Items	Median	Mean	Skewness	Kurtosis	SD	IQR	Factor Loading
4. Fidgety, unable to sit still	1	0.78	0.39	1.80	0.76	[0–1]	0.65
8. Daydreams too much	0	0.67	0.62	2.01	0.75	[0–1]	0.60
9. Distracted easily	1	0.91	0.14	1.76	0.76	[0–1]	0.78
14. Has trouble concentrating	0	0.44	1.21	3.20	0.67	[0–1]	0.86
11. Feels sad, unhappy	0	0.44	1.20	3.22	0.66	[0–1]	0.77
13. Feels hopeless	1	0.76	0.43	1.88	0.75	[0–1]	0.84
19. Is down on him or herself	0	0.48	0.97	2.84	0.64	[0–1]	0.73
22. Worries a lot	0	0.53	0.96	2.61	0.70	[0–1]	0.62
27. Seems to be having less fun	1	0.79	0.37	1.83	0.76	[0–1]	0.72
16. Fights with others	0	0.48	1.06	2.88	0.67	[0–1]	0.73
29. Does not listen to rules	0	0.60	0.69	2.36	0.68	[0–1]	0.68
31. Does not understand other people’s feelings	1	0.68	0.56	2.10	0.72	[0–1]	0.55
32. Teases others	0	0.45	1.04	3.04	0.61	[0–1]	0.73
33. Blames others for his or her troubles	0	0.25	2.02	6.11	0.53	[0–0]	0.79
34. Takes things that do not belong to him or her	0	0.25	2.06	6.30	0.53	[0–0]	0.76
35. Refuses to share	0	0.51	1.02	2.71	0.70	[0–1]	0.54

Note: SD = Standard Deviation; IQR = Interquartile range.

### Confirmatory factor analysis and reliability of PSC

All goodness of fit indicators from the confirmatory factor analysis had a good or acceptable fit, for both the original versions of PSC (including the item "#7, Acts as if driven by motor") and the new PSC versions, with very small differences in performance in favor of the new PSC versions (Table 4).

**Table 4:** Model fit indices of CFA.

Indicator	PSC-35-Y	PSC-34-Y	PSC-17-Y	PSC-16-Y	Good fit	Acceptable fit
RMSEA	0.06	0.06	0.05	0.04	≤0.05	≤0.08
SRMR	0.07	0.07	0.06	0.05	≤0.05	≤0.10
NFI	0.95	0.96	0.98	0.98	≥0.95	≥0.90
NNFI	0.96	0.96	0.98	0.98	≥0.97	≥0.95
CFI	0.96	0.96	0.98	0.99	≥0.97	≥0.90
GFI	0.97	0.97	0.99	0.99	>0.95	>0.90
AGFI	0.96	0.96	0.98	0.98	>0.90	>0.90
CHISQ/df	5.99	5.76	4.70	4.32		

Regarding reliability, all subscales had an acceptable omega coefficient in all of the questionnaire versions, except for the Attention subscale of the PSC-17-Y, which had an omega value of 0.67 (Table 5).

**Table 5:** PSC reliability.

Dimension	Omega
PSC-35-Y	0.78
PSC-34	0.78
PCS-17-Y	0.93
*Attention*	0.67
*Internalizing*	0.79
*Externalizing*	0.78
PCS-16-Y	0.94
*Attention*	0.77
*Internalizing*	0.79
*Externalizing*	0.78

### Concurrent validity with SDQ questionnaire

All PSC-16-Y subscales had a weak, although significant, correlation with all of the SDQ subscales. The Attention subscale had a higher correlation with the Hyperactivity subscale of the SDQ. The Internalizing subscale had a higher correlation with the Emotional Symptoms subscale of SDQ (0.38). The Externalizing subscale had a higher correlation with the Conduct Problems subscale of SDQ (0.39). All three PSC-16-Y subscales had a negative correlation with the Prosocial Behavior subscale of SDQ (Table 6).

**Table 6:** Spearman correlation between PSC-16-Y and SDQ.

Subscales	PSC—Attention	PSC—Internalizing	PSC—Externalizing
SDQ–Emotional Symptoms	0.22	0.38	0.17
SDQ–Conduct Problems	0.22	0.26	0.39
SDQ–Hyperactivity	0.44	0.17	0.24
SDQ–Peer Problems	0.10	0.27	0.21
SDQ–Prosocial Behavior	-0.11	-0.09	-0.26

Note: All of the correlations had a p-value <0.0001.

### School factors associated with PSC-16-Y

Regarding bullying experience, the strongest association with the PSC-16-Y total score was bullying perpetration (β = 3.19, p<0.001). Victimization was highly associated with Internalizing subscale score (β = 1.02, p<0.001), and the score in the perpetration subscale was highly associated with Externalizing subscale score (β = 1.50, p<0.001).

A better school climate and a stronger school membership were negatively associated with the PSC-16-Y total score, and with all subscales (Table 7A–7D).

**Table 7:** a: Risk and protective factors associations with PSC-16-Y. b: Risk and protective factors associations with Attention subscale score of PSC-16-Y. c: Risk and protective factors associations with Internalizing subscale score of PSC-16-Y. d: Risk and protective factors associations with Externalizing subscale score of PSC-16-Y.

Variables	PSC-16-Y Score				
	β*	[95% CI]	P-value	R-squared	Adj R-squared
**Bullying experience**					
Bullying victimization	2.55	[2.11–2.99]	<0.001	0.0510	0.0501
Bullying perpetrator	3.19	[2.76–3.63]	<0.001	0.0726	0.0718
Bullying bystander	0.62	[0.22–1.03]	0.003	0.0153	0.0143
**Others school factors**					
School climate	-0.45	[-0.49- -0.40]	<0.001	0.1085	0.1077
Sense of school membership	-0.16	[-0.18- -0.14]	<0.001	0.0914	0.0906
**Variables**	**Attention subscale score**				
	β*	[95% CI]	P-value	R-squared	Adj R-squared
**Bullying experience**					
Bullying victimization	0.64	[0.49–0.79]	<0.001	0.0254	0.0246
Bullying perpetrator	0.80	[0.64–0.96]	<0.001	0.0332	0.0324
Bullying bystander	0.30	[0.15–0.45]	<0.001	0.0106	0.0098
**Others school factors**					
School climate	-0.10	[-0.12- -0.08]	<0.001	0.0419	0.0410
Sense of school membership	-0.04	[-0.05- -0.03]	<0.001	0.0400	0.0391
**Variables**	**Internalizing subscale score**				
	β*	[95% CI]	P-value	R-squared	Adj R-squared
**Bullying experience**					
Bullying victimization	1.02	[0.85–1.18]	<0.001	0.0507	0.0499
Bullying perpetrator	0.76	[0.58–0.94]	<0.001	0.0284	0.0275
Bullying bystander	0.14	[-0.02–0.31]	0.083	0.0086	0.0078
**Others school factors**					
School climate	-0.19	[-0.21- -0.17]	<0.001	0.1085	0.1078
Sense of school membership	-0.07	[-0.07- -0.06]	<0.001	0.0891	0.0883
**Variables**	**Externalizing subscale score**				
	β*	[95% CI]	P-value	R-squared	Adj R-squared
**Bullying experience**					
Bullying victimization	0.67	[0.47–0.88]	<0.001	0.0306	0.0297
Bullying perpetrator	1.50	[1.30–1–69]	<0.001	0.0795	0.0787
Bullying bystander	0.11	[-0.07–0.30]	0.227	0.0189	0.0181
**Others school factors**					
School climate	-0.15	[-0.17- -0.12]	<0.001	0.0660	0.0652
Sense of school membership	-0.05	[-0.06- -0.04]	<0.001	0.0602	0.0594

Note: All analyses were adjusted using age and gender variables. β* = Unstandardized Beta coefficient.

## Discussion

To our knowledge, this is the first validation study of the Spanish version of the PSC-Y questionnaire among early adolescents in Latin America. Our findings support the decision to eliminate item #7, "Acts as if driven by motor" to improve the psychometric properties of the instruments. The resulting PSC-Y questionnaires, PSC-34-Y and PSC-16-Y, had a good structure of the items. The construct validation indicators using confirmatory factor analysis showed that all of the new subscales had good or adequate goodness of fit adjustment. We confirmed the original three-factor structure of the PSC questionnaire (Attention, Internalizing, and Externalizing subscales) [38], and there was a good correlation between the PSC-16-Y total score and its subscales and the subscales of the SDQ [19]. On the other hand, the reliability was good or acceptable for the total scale and for all subscales.

We decided to eliminate the item "Acts as if driven by motor" because the psychometric analyses indicated that this statement was, very likely, rather understood as a positive feature by adolescents instead of considering it as a negative behavior. In the translation and adaptation process, we explored several Spanish versions of the item, including the version proposed by authors ("Eres incansable"), and, in all the cases, the statement was considered as a positive behavior. Additionally, the literal translation of the item does not have a clear meaning in Spanish. Therefore, it is advisedly to eliminate this item, especially if we also consider that the Attention subscale, where this item belongs, already has a related item ("Fidgety, unable to sit still") which captures the same problematic symptom that "Acts as if driven by motor" intents to assess.

There are much fewer validation studies of the PSC-Y, 35 items, and PSC-17-Y (self-reported), compared with the number of studies exploring the validity of PSC, 35 items, and PSC-17 (parent/caregiver-reported). For instance, in one study [61] using the PSC-Y (35 items) to detect high-risk adolescents, it was found that this population had a higher likelihood of seeking help, a higher prevalence of previous mental health treatment, and a higher chance of coming from low-income families. Therefore, the PSC helped to identify relevant features of the population most at risk. Another study in the USA [62], using the PSC (35 items), reported good concurrent validity of the self-reported instrument with the teacher and parent-reported versions of the PSC. However, the reliability, using a test-retest procedure, was only acceptable (kappa of 0.50). Different contextual and cultural variables may affect the performance of this instrument, supporting the need for local studies of its validation.

On the other hand, regarding the PSC-17-Y, one study [63] found a good Cronbach’s alpha (alpha = 0.78) for the total scale, but the reliability of its subscales was not reported. Finally, in another study [64] on the child welfare population, it was found that internalizing subscale was strongly correlated, the attention subscale was moderately correlated, and externalizing subscale was weakly correlated with the Screen for Child Anxiety Related Disorders scales.

All these studies can be seen as complementary to each other; however, the scarce number of studies, limited information reported on the psychometric features, and the specificity of the population studied, justify the need for our study and the importance of the findings in a general population of adolescents.

Finally, there are only three other studies exploring the validity of the PSC questionnaire in Chile, but in all cases, they used the parent version of the PSC. The first study assessed the concurrent validity of the PSC (35 items), using the Teacher Observation of Classroom Adaptation–Revised (TOCA-R), and its four factors. The correlations between the total score of PSC and the four factors were acceptable (between 0.126 and 0.4399 [31]. Nevertheless, Cronbach’s alpha of the total score was good (0.853). Another study explored the structure of PSC (35 items); however, it did not find the proposed three-factor structure but a six-factor structure. They also explored the concordance of these six factors with the psychiatric diagnosis using the Schedule for Affective Disorders and Schizophrenia for School-Age Children-Present and Lifetime version (Kiddie-SADS-PL), showing that only half of the cases had a good concordance, but the sample was small to reach clear conclusions [33]. Finally, a third study [32] explored the concurrent validity of the PSC-17 with the TOCA-R, finding moderate correlations between them [32,54]. In the same study, the reliability for the subscales was 0.72 for the externalizing subscale, 0.76 for the internalizing subscale, and 0.61 for the attention subscale. This study also performed a confirmatory factor analysis finding the three-factor structure of the PSC, similar to our study.

Among the strengths of our study, we can mention the following: we surveyed a large sample size (N = 3,968); the validation was explored in both versions of the PSC-Y (35 and 17 items); and we performed several statistical analyses in order to have a wide range to sources of validation, such as confirmatory factor analyses and Spearman’s correlation with another validated questionnaire. In addition, we explored the associations between different risk and protective factors and the PSC-Y-16 subscales as outcomes. These questionnaires can be administered easily in school settings which may help to identify students at risk and refer to proper early intervention or treatment in health services if needed.

This study has some limitations. First, this was a self-reported questionnaire, so information bias may exist, especially related to social desirability. Second, we only included students attending schools with high socioeconomic vulnerability, so the results may not represent adolescents from more affluent families. However, most students in Chile attend schools with high socioeconomic vulnerability; therefore, the results can be generalizable to most of the school population in Chile and perhaps in Latin America. Third, given the proposed changes in the content of the questionnaire in the Spanish version studied here compared with the original English version, the cross-cultural comparisons in the prevalence of mental health problems among adolescents should be made with caution. And finally, this was a cross-sectional study, so no causality can be concluded regarding the associations.

In all, the Spanish version of the PSC-Y questionnaire has good indicators of its validity in Chile, and our study reduces the knowledge gap of this information among early adolescents. This questionnaire may be used by the Government of Chile, other Latin American countries, and health and school professionals to identify at-risk populations. Future research also needs to determine the cutoff score in order to better detect adolescents who need help and to find other personal and contextual variables associated with mental health problems in order to plan preventive interventions.

## Supporting information

S1 File (PDF)

S2 File English version of PSC-Y Questionnaire. (DOCX)

S3 File English version of PSC-17-Y Questionnaire. (DOCX)

S4 File CFA descriptive Statistics of PSC. (DOCX)

S5 File Subscales medians of PSC. (DOCX)
