# Supplementary material for: Validation of the Spanish version of the Pediatric Symptom Checklist (PSC) to identify and assess psychosocial problems among early adolescents in Chile
Source: PLoS One. 2023 Apr 6;18(4):e0283921. doi: 10.1371/journal.pone.0283921 (PMC10079088; doi:10.1371/journal.pone.0283921)
Supplement: S2 File — (DOCX) [file pone.0283921.s002.docx]

Pediatric Symptom Checklist - Youth Report (Y-PSC)

Please mark under the heading that best fits you:

1. Complain of aches or pains............................

2. Spend more time alone.................................

Never Sometimes Often

3. Tire easily, little energy............................

4. Fidgety, unable to sit still..........................

1. Have trouble with teacher………………
2. Less interested in school.…………... 7. Act as if driven by motor............................. 8. Daydream too much..................................... 9. Distract easily....................................... 10. Are afraid of new situations.......................... 11. Feel sad, unhappy..................................... 12. Are irritable, angry.................................. 13. Feel hopeless......................................... 14. Have trouble concentrating............................ 15. Less interested in friends............................ 16. Fight with other children.............................
3. Absent from school. …………………….
4. School grades dropping. ……………….. 19. Down on yourself......................................

20. Visit doctor with doctor finding nothing wrong........ 21. Have trouble sleeping................................. 22. Worry a lot...........................................

23. Want to be with parent more than before............... 24. Feel that you are bad................................. 25. Take unnecessary risks................................ 26. Get hurt frequently................................... 27. Seem to be having less fun............................

28. Act younger than children your age.................... 29. Do not listen to rules................................ 30. Do not show feelings..................................

31. Do not understand other people's feelings............. 32. Tease others..........................................

33. Blame others for your troubles........................

34. Take things that do not belong to you................. 35. Refuse to share......................................

Reprinted under a CC BY license, with permission from [Michael Jellinek], originally published in [1986] under a CC-BY-NC license
