# Supplementary material for: Validation of the Spanish version of the Pediatric Symptom Checklist (PSC) to identify and assess psychosocial problems among early adolescents in Chile
Source: PLoS One. 2023 Apr 6;18(4):e0283921. doi: 10.1371/journal.pone.0283921 (PMC10079088; doi:10.1371/journal.pone.0283921)
Supplement: S3 File — (DOCX) [file pone.0283921.s003.docx]

# Name: Record #:

| **YOUTH** | **PEDIATRIC** | **SYMPTOM** | **CHECKLIST-17** | **(Y** | **PSC-17)** |  |
| --- | --- | --- | --- | --- | --- | --- |

# Date of Birth: Today’s Date:

| **Please mark under the heading that best fits you:** | | | **NEVER** | | **SOMETIMES** | | | **OFTEN** |
| --- | --- | --- | --- | --- | --- | --- | --- | --- |
|  | **Fidgety, unable to sit still** |  |  | **0** |  | **1** |  | **2** |
|  | **Feel sad, unhappy** |  |  | **0** |  | **1** |  | **2** |
|  | **Daydream too much** |  |  | **0** |  | **1** |  | **2** |
|  | **Refuse to share** |  |  | **0** |  | **1** |  | **2** |
|  | **Do not understand other people’s feelings** |  |  | **0** |  | **1** |  | **2** |
|  | **Feel hopeless** |  |  | **0** |  | **1** |  | **2** |
|  | **Have trouble concentrating** |  |  | **0** |  | **1** |  | **2** |
|  | **Fight with other children** |  |  | **0** |  | **1** |  | **2** |
|  | **Down on yourself** |  |  | **0** |  | **1** |  | **2** |
|  | **Blame others for your troubles** |  |  | **0** |  | **1** |  | **2** |
|  | **Seem to be having less fun** |  |  | **0** |  | **1** |  | **2** |
|  | **Do not listen to rules** |  |  | **0** |  | **1** |  | **2** |
|  | **Act as if driven by a motor** |  |  | **0** |  | **1** |  | **2** |
|  | **Tease others** |  |  | **0** |  | **1** |  | **2** |
|  | **Worry a lot** |  |  | **0** |  | **1** |  | **2** |
|  | **Take things that do not belong to you** |  |  | **0** |  | **1** |  | **2** |
|  | **Distract easily** |  |  | **0** |  | **1** |  | **2** |

OFFICE USE ONLY

Total Total Total Grand Total + +

Form adapted with permission for *Feelings Need Check Ups Too*, 2004 ©1988, M. Jellinek & J.M. Murphy, Massachusetts General Hospital (PSC-17 created by W. Gardner & K. Kelleher) and Bright Futures in Practice: Mental Health, 2002. Reprinted under a CC BY license, with permission from [Michael Jellinek], originally published in [1986] under a CC-BY-NC license
