# Supplementary material for: Validation of the Spanish version of the Pediatric Symptom Checklist (PSC) to identify and assess psychosocial problems among early adolescents in Chile
Source: PLoS One. 2023 Apr 6;18(4):e0283921. doi: 10.1371/journal.pone.0283921 (PMC10079088; doi:10.1371/journal.pone.0283921)
Supplement: S4 File — (DOCX) [file pone.0283921.s004.docx]

S3 Descriptive Statistics and loading CFA of the PSC-35-Y.

| Items | Median | Mean | Skewness | Kurtosis | SD | IQR | Factor Loading |
| --- | --- | --- | --- | --- | --- | --- | --- |
| Complain of aches or pains | 0 | 0.53 | 0.8 | 2.59 | 0.64 | [0 - 1] | 0.47 |
| Spend more time alone | 0 | 0.39 | 1.27 | 3.56 | 0.6 | [0 - 1] | 0.59 |
| Tire easily, little energy | 0 | 0.54 | 0.85 | 2.57 | 0.67 | [0 - 1] | 0.54 |
| Fidgety, unable to sit still | 1 | 0.76 | 0.44 | 1.84 | 0.76 | [0 - 1] | 0.55 |
| Have trouble with teacher | 0 | 0.39 | 1.28 | 3.57 | 0.6 | [0 - 1] | 0.59 |
| Less interested in school | 0 | 0.55 | 0.83 | 2.54 | 0.67 | [0 - 1] | 0.49 |
| Act as if driven by motor | 1 | 1.23 | -0.4 | 1.86 | 0.75 | [1 - 2] | -0.03 |
| Daydreams too much | 0 | 0.65 | 0.66 | 2.09 | 0.74 | [0 - 1] | 0.5 |
| Distracted easily | 1 | 0.9 | 0.17 | 1.7 | 0.77 | [0 - 2] | 0.66 |
| Are afraid of new situations | 1 | 0.64 | 0.65 | 2.2 | 0.71 | [0 - 1] | 0.54 |
| Feels sad, unhappy | 0 | 0.45 | 1.15 | 3.09 | 0.66 | [0 - 1] | 0.73 |
| Are irritable, angry | 0 | 0.58 | 0.79 | 2.36 | 0.71 | [0 - 1] | 0.65 |
| Feel hopeless | 0 | 0.43 | 1.23 | 3.32 | 0.64 | [0 - 1] | 0.76 |
| Have trouble concentrating | 1 | 0.76 | 0.41 | 1.94 | 0.73 | [0 - 1] | 0.71 |
| Less interested in friends | 0 | 0.4 | 1.33 | 3.54 | 0.64 | [0 - 1] | 0.6 |
| Fight with other children | 0 | 0.47 | 0.96 | 2.87 | 0.62 | [0 - 1] | 0.61 |
| Absent from school | 1 | 0.63 | 0.41 | 2.33 | 0.61 | [0 - 1] | 0.36 |
| School grades dropping | 1 | 0.62 | 0.62 | 2.32 | 0.67 | [0 - 1] | 0.55 |
| Down on yourself | 0 | 0.5 | 1.02 | 2.77 | 0.68 | [0 - 1] | 0.71 |
| Visit doctor with doctor finding nothing wrong | 0 | 0.51 | 1.02 | 2.68 | 0.71 | [0 - 1] | 0.39 |
| Have trouble sleeping | 0 | 0.56 | 0.87 | 2.42 | 0.72 | [0 - 1] | 0.59 |
| Worry a lot | 1 | 0.79 | 0.36 | 1.88 | 0.74 | [0 - 1] | 0.52 |
| Want to be with parent more than before | 1 | 1.12 | -0.21 | 1.62 | 0.79 | [0 - 2] | 0.34 |
| Feel that you are bad | 0 | 0.52 | 0.96 | 2.67 | 0.68 | [0 - 1] | 0.71 |
| Take unnecessary risks | 0 | 0.46 | 1.1 | 3.03 | 0.65 | [0 - 1] | 0.65 |
| Get hurt frequently | 0 | 0.52 | 0.97 | 2.63 | 0.7 | [0 - 1] | 0.58 |
| Seem to be having less fun | 0 | 0.46 | 1.14 | 3.07 | 0.66 | [0 - 1] | 0.68 |
| Act younger than children your age | 0 | 0.55 | 0.9 | 2.45 | 0.72 | [0 - 1] | 0.52 |
| Do not listen to rules | 0 | 0.59 | 0.71 | 2.41 | 0.67 | [0 - 1] | 0.6 |
| Do not show feelings | 1 | 0.71 | 0.51 | 2.01 | 0.73 | [0 - 1] | 0.48 |
| Do not understand other people’s feelings | 1 | 0.65 | 0.62 | 2.19 | 0.71 | [0 - 1] | 0.42 |
| Tease others | 0 | 0.47 | 0.97 | 2.89 | 0.62 | [0 - 1] | 0.61 |
| Blame others for your troubles | 0 | 0.27 | 1.96 | 5.8 | 0.55 | [0 - 0] | 0.62 |
| Take things that do not belong to you | 0 | 0.25 | 2.03 | 6.23 | 0.52 | [0 - 0] | 0.6 |
| Refuses to share | 0 | 0.48 | 1.08 | 2.9 | 0.68 | [0 - 1] | 0.46 |
