# Supplementary material for: Validation of the Spanish version of the Pediatric Symptom Checklist (PSC) to identify and assess psychosocial problems among early adolescents in Chile
Source: PLoS One. 2023 Apr 6;18(4):e0283921. doi: 10.1371/journal.pone.0283921 (PMC10079088; doi:10.1371/journal.pone.0283921)
Supplement: S5 File — (DOCX) [file pone.0283921.s005.docx]

S4 Subscales medians from PSC-16-Y

|  | N | Total PSC16 | Attention | Internalizing | Externalizing |
| --- | --- | --- | --- | --- | --- |
| SDQ – Total | | | | | |
| Normal $(0-15)$ | 1.172 | 6 | 2 | 1 | 2 |
| Borderline $(16-19)$ | 357 | 12 | 4 | 3 | 4 |
| Abnormal $(20-40)$ | 399 | 14 | 4 | 4 | 5 |
| p value | | <0.0001 | <0.0001 | <0.0001 | <0.0001 |
| SDQ – Emotional Symptoms | | | | | |
| Normal $(0-5)$ | 1.432 | 7 | 3 | 2 | 2 |
| Borderline $(6)$ | 195 | 11 | 4 | 4 | 3 |
| Abnormal $(7-10)$ | 301 | 13 | 4 | 5 | 4 |
| p value | | <0.0001 | <0.0001 | <0.0001 | <0.0001 |
| SDQ – Conduct Problems | | | | | |
| Normal $(0-3)$ | 1.291 | 6 | 3 | 2 | 2 |
| Borderline $(4)$ | 239 | 11 | 4 | 3 | 4 |
| Abnormal $(5-10)$ | 400 | 13 | 4 | 4 | 5 |
| p value | | <0.0001 | <0.0001 | <0.0001 | <0.0001 |
| SDQ – Hyperactivity | | | | | |
| Normal $(0-5)$ | 1.331 | 6 | 2 | 2 | 2 |
| Borderline $(6)$ | 233 | 11 | 4 | 3 | 3 |
| Abnormal $(7-10)$ | 366 | 13 | 5 | 3 | 4 |
| p value | | <0.0001 | <0.0001 | <0.0001 | <0.0001 |
| SDQ – Peer Problems | | | | | |
| Normal $(0-3)$ | 1.246 | 7 | 3 | 2 | 2 |
| Borderline $(4-5)$ | 478 | 10 | 4 | 3 | 4 |
| Abnormal $(6-10)$ | 206 | 13 | 3 | 4 | 4 |
| p value | | <0.0001 | <0.0001 | <0.0001 | <0.0001 |
| SDQ – Prosocial Behaviour | | | | | |
| Normal $(0-4)$ | 1.532 | 8 | 3 | 2 | 2 |
| Borderline $(5)$ | 205 | 10 | 3 | 2 | 4 |
| Abnormal $(6-10)$ | 193 | 10 | 3 | 2 | 4 |
| p value | | <0.0001 | 0.43 | 0.36 | <0.0001 |
